# Interaction of the Host and Viral Genome and Their Influence on HIV Disease

**DOI:** 10.3389/fgene.2018.00720

**Published:** 2019-01-23

**Authors:** Riley H. Tough, Paul J. McLaren

**Affiliations:** ^1^JC Wilt Infectious Diseases Research Centre, Public Health Agency of Canada, Winnipeg, MB, Canada; ^2^Department of Medical Microbiology and Infectious Diseases, University of Manitoba, Winnipeg, MB, Canada

**Keywords:** HIV infection, genetic variation, set point viral load, genome-wide studies, genetic epidemiology, host–pathogen interaction

## Abstract

The course of Human Immunodeficiency Virus type 1 (HIV) infection is a dynamic interplay in which both host and viral genetic variation, among other factors, influence disease susceptibility and rate of progression. HIV set-point viral load (spVL), a key indicator of HIV disease progression, has an estimated 30% of variance attributable to common heritable effects and roughly 70% attributable to environmental factors and/or additional non-genetic factors. Genome-wide genotyping and sequencing studies have allowed for large-scale association testing studying host and viral genetic variants associated with infection and disease progression. Host genomics of HIV infection has been studied predominantly in Caucasian populations consistently identifying human leukocyte antigen (HLA) genes and C-C motif chemokine receptor 5 as key factors of HIV susceptibility and progression. However, these studies don’t fully assess all classes of genetic variation (e.g., very rare polymorphisms, copy number variants etc.) and do not inform on non-European ancestry groups. Additionally, viral sequence variability has been demonstrated to influence disease progression independently of host genetic variation. Viral sequence variation can be attributed to the rapid evolution of the virus within the host due to the selective pressure of the host immune response. As the host immune system responds to the virus, e.g., through recognition of HIV antigens, the virus is able to mitigate this response by evolving HLA-specific escape mutations. Diversity of viral genotypes has also been correlated with moderate to strong effects on CD4+ T cell decline and some studies showing weak to no correlation with spVL. There is evidence to support these viral genetic factors being heritable between individuals and the evolution of these factors having important consequences in the genetic epidemiology of HIV infection on a population level. This review will discuss the host-pathogen interaction of HIV infection, explore the importance of host and viral genetics for a better understanding of pathogenesis and identify opportunities for additional genetic studies.

## Introduction

Since the discovery of Human Immunodeficiency Virus type 1 (HIV) in the 1980s, a major goal of the infectious disease research community has been to study the pathogenesis of HIV disease to guide the development of therapeutics and, more recently, a functional cure. Since the start of the epidemic there have been an estimated 77.3 million (59.9–100 million) individuals infected with HIV and, in 2017, an estimated 36.9 million (31.1–43.9 million) individuals living with HIV globally (UNAIDS, 2018). More than 25 therapeutics have been developed for the treatment of HIV, although there is still no preventative vaccine and no functional cure (Tseng et al., 2015). The use of combination antiretroviral therapy (cART) has been shown to drastically improve the longevity and quality of life in people living with HIV infection.

While the vast majority of the infected population requires cART to achieve suppressed viral load, it has become widely accepted that some individuals are able to maintain suppression without the use of cART (reviewed in Deeks and Walker, 2007) and those who are entirely resistant to infection (Horton et al., 2010). The significance of a suppressed viral load was underscored in 2016 when it was determined that an undetectable viral load significantly reduced the risk of HIV transmission which has led to the statement “undetectable equals untransmittable” (U = U) (Cohen et al., 2011; Rodger et al., 2016). Therefore, achieving viral suppression in the majority of the infected population has become a major goal in ending the HIV epidemic. This is the motivation behind the UNAIDS (90-90-90) initiative which aims to have 90% of the global infected population diagnosed for HIV, 90% of diagnosed individuals on appropriate treatment, and 90% of individuals on treatment having viral suppression (UNAIDS, 2018). Meeting this goal by the year 2020 is a major ongoing effort by the global community with the hopes of ending the AIDS epidemic by 2030. It is likely that both existing and novel anti-HIV interventions will be required to meet this goal.

In this review, we will outline new advances in HIV host and viral genetic/genomic studies and discuss how genetic variability can modify susceptibility, disease progression and the dynamics of the host–pathogen interaction. We will also identify gaps in the current HIV genomics research and opportunities for future investigations.

## Background on HIV and Disease Progression

### HIV Disease Progression

**FIGURE 1:**
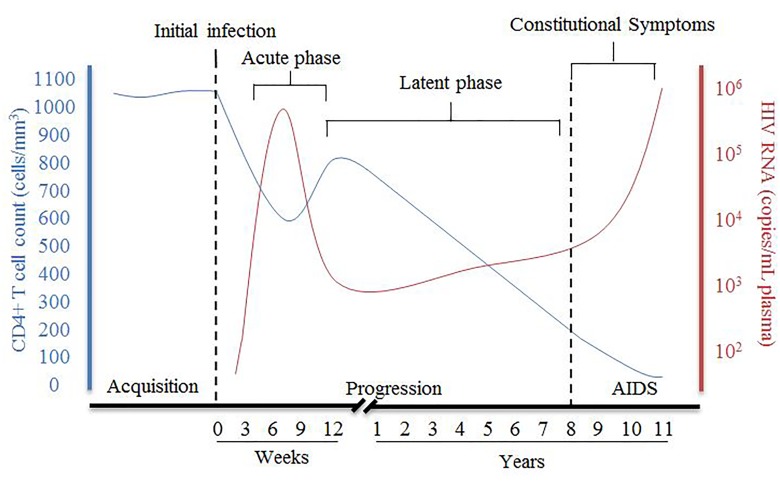
Summary of pathogenesis of HIV infection in the presence of combined antiretroviral therapy. The disease progression of HIV in this figure is represented by the relative CD4+ T cell count in cells/mm^3^ (blue) and HIV viral RNA in copies/mL plasma (red) and their relationship during HIV from acquisition to the development of AIDS [Figure was adapted from An and Winkler (2010)].

HIV disease progression consists of an acute phase where, after the initial infection, there is a peak of viral RNA and a drastic decrease of host CD4+ T cells (Figure 1), which can be accompanied with flu-like symptoms. The acute phase generally corresponds to a high viral titer and therefore increased transmissibility so early detection of individuals suspected of infection is important. Following acute infection, there is a brief recovery phase where there is some recovery of CD4+ T cells and a decrease of viral RNA, but then progressing into a persistent decrease of CD4+ T cells and increase of viral RNA associated with chronic stages of infection. The chronic phase can last over 10 years before an individual develops acquired immunodeficiency syndrome (AIDS) although rate of progression can vary dramatically. AIDS is defined as a CD4+ T cell count of less than 200 cells/mm^3^, taken from an individual living with HIV.

During chronic, untreated infection, the amount of viral RNA in blood can remain relatively constant in an individual and is referred to as the set-point viral load (spVL). SpVL can change quite drastically between individuals but has been shown to be relatively constant within a person with a higher viral load being strongly associated with a faster disease progression (Mellors et al., 1996; O’Brien et al., 1996). Rate of disease progression is also variable in the infected population, with the majority of untreated individuals progressing to AIDS in 5–10 years (Figure 1). Although research has shown there are some viral and host factors which can influence the rate of progression and spVL of an individual, much of the variance in these traits remains unknown (McLaren et al., 2015).

High spVL is a public health concern due to increased transmission risk in all risk groups including intravenous drug users, men who have sex with men (MSM), and heterosexual transmission (Crepaz et al., 2016). Although most individuals undergo viral suppression during ART, small populations of individuals, called HIV controllers, are able to achieve this suppression in the absence of therapy (Cao et al., 1995). There is substantial variance in viremia between HIV controllers and progressors, as well as rate of disease progression between the two groups. There have also been several observations of both extreme long-term non-progression and extreme rapid progression, although their definitions are not consistently held (Gurdasani et al., 2014; Olson et al., 2014). HIV controllers and progressors have been important populations of study to determine the effects of viral, host, and environmental factors which contribute to the variance in viral load and disease progression.

The ability of an individual to suppress their viral load, in the absence or with the assistance of cART, can significantly decrease HIV disease progression and greatly improve quality of life. This phenomenon has warranted a large amount of time to research the variability of HIV disease progression and has resulted in a wealth of knowledge regarding the complex interaction of HIV and host proteins. There are many factors which impact HIV disease progression such as viral and host genetic diversity, host–pathogen interaction and environmental factors. Although the root causes of variability in spVL and rates of progression are not fully understood, one area that has received significant attention, and produced significant discoveries, is the role of viral and host genetic variability, and the dynamics of host–pathogen interaction.

### Methods for Studying Genetic Diversity

Genome-wide genotyping and sequencing studies have marked a shift in the direction of human genetic research from the use of candidate gene studies to genome-wide approaches allowing for the identification of large number of genetic variants associated with control of HIV infection. Genome-wide association studies (GWAS) provide an unbiased scan of the genome for common single nucleotide polymorphisms (SNPs) and small insertions and deletions (indels) associated with a particular trait (e.g., viral load). While these types of studies do not provide any causal information, they direct researchers to a region of interest which requires further studies to determine causality. However, GWAS require strict statistical standards and require large sample sizes which can make study designs difficult (Ioannidis et al., 2011).

## HIV Genetic Impact on HIV Pathogenesis

### HIV Viral Diversity

The global HIV pandemic originated from the independent zoonotic transmission events between non-human primates and humans which resulted in four different groups of HIV M, N, O, and P (Keele et al., 2006; reviewed in Taylor et al., 2008; Vallari et al., 2011). HIV group M, which is responsible for the majority of global infections, can be separated into eight genetically distinct subtypes: A, B, C, D, F, G, H, J, and K with additional circulating recombinant forms (CRFs) that have also been observed. Due to the high error rate of the reverse transcriptase (RT) enzyme and the rapid replication rate of HIV, the virus is able to generate large numbers of mutations within its genome (Fryer et al., 2010; Cuevas et al., 2015; Roberts et al., 2016). These viral mutations, if beneficial for survival, can create quasispecies that are resistant to immune responses (Crawford et al., 2009; Bronke et al., 2013) and antiretroviral therapy (Roche et al., 2011; Hayashida et al., 2016). If these mutations enhance the viruses’ ability to circumvent cART or host defense mechanisms, they are termed escape mutations. Escape mutations may be at a cost of viral fitness resulting in a more resistant virus but with decreased virulence within the host (Fryer et al., 2012; Song et al., 2012; Shahid et al., 2015). There have also been reports of specific escape mutations causing decreased viral fitness during transmission to a new host (Chopera et al., 2008). While the host immune system exerts strong selective pressure on HIV to develop escape mutations, evidence shows that this process is slowed in the presence of cART (Knapp et al., 2012).

### Viral Sequencing

Since HIV has such a high mutation rate, viral sequencing is an essential process for determining potential drug resistance at the beginning of treatment. Early detection of drug resistance is necessary to help guide treatments options which are most suitable for a particular patient. This process is traditionally performed by sequencing plasma HIV RNA and looking for known variants in the RT, protease (PR), and integrase (IN) genes that are associated with resistance. Recently, the field of HIV sequencing has started a shift from traditional Sanger sequencing methods toward next generation sequencing (NGS) for the detection of low frequency viral variants. While NGS is able to detect more variants than traditional RNA or DNA Sanger sequencing, the clinical relevance of low frequency viral variants still requires more investigation before use in a clinical setting (Alidjinou et al., 2017; Inzaule et al., 2018). Particularly, HIV exists as a number of quasispecies within a host resulting in a large amount of intra-host genetic diversity which can make interpretation of sequencing results challenging.

### Impact of HIV Viral Subtype and Sequence Diversity on Pathogenesis

Viral sequence diversity has been shown to affect disease progression independently of host factors (Pai et al., 2012). Current evidence suggests subtype D is associated with faster disease progression when compared to subtype A. In Africa, subtype D had a faster disease progression than subtype A during the pre-ART period (Vasan et al., 2006; Kiwanuka et al., 2008; Ssemwanga et al., 2013). In a large study using data from the CASCADE collection (3364 seroconverting individuals of known subtype), they observed slower CD4+ T cell decline in individuals infected by subtypes A, C, and CRF02 compared to those infected with subtype B (Touloumi et al., 2013). However, in an analysis adjusting for demographic factors there was no significant difference in time to AIDS or median viral load set point between individuals infected by different subtypes. The results of the study also show that recombinant CRF01 and CRF02 are not more virulent than parent isolates (Touloumi et al., 2013).

Investigations of within subtype diversity have also identified some differences in rates of progression. In a study looking at 8483 United Kingdom patients prior to antiretroviral therapy, genetic diversity in the polymerase gene explained roughly 5.7% of the variance of spVL in subtype B infected individuals (Hodcroft et al., 2014). More recently, studies of near full-length HIV sequences in the Swiss HIV Cohort Study have suggested that the viral genome can explain up to 30% of the observed variance in spVL (Bartha et al., 2017; Bertels et al., 2018). Additional studies in clade B and across multiple clades, have resulted in similar heritability estimates with viral genotype explaining between 26 and 44% of observed variance in spVL (reviewed in Bonhoeffer et al., 2015).

While viral sequence variation can have a direct impact on disease progression causing increased or decreased viral load, this may be reflecting host genetic pressure on spVL as variation of viral sequences can be attributed to escape mutations. In a joint host/viral genetic analysis, 23.6% of the variability in HIV spVL mapped to known epitope regions, suggesting that this variability is the result of pathogen evolution away from host HLA (Bartha et al., 2017). Furthermore, the authors showed that by not accounting for viral genetics that reliability of heritability estimates by host genetic studies may be impacted.

At the population level, adaptation of the viral genome to host HLA can impact the rate of disease progression to prevent detection and elimination of the virus by CD8+ T cells. There are many HLA alleles which have been associated with control of HIV infection. HLA-B^∗^57:01 is known to strongly decrease the rate of disease progression, however, across multiple continents; it has been shown that HIV is developing an escape mutation, I135X, which attenuates control (Kawashima et al., 2009). Similarly, the protective allele HLA-B^∗^52:01-C^∗^12:02 is associated with decreased viral load in Japan due to the development of escape mutations in the *pol* gene of HIV which causes poor viral fitness (Murakoshi et al., 2017). These escape mutations allow the virus to evade HLA detection and change the dynamics of disease progression, albeit at the cost of viral fitness. Additional studies in Africa (Payne et al., 2014) and North America (Brumme et al., 2018) have also demonstrated local adaptation of HIV to common HLA alleles, with a potential impact on disease progression rates in the population.

While the impact of HIV sequence variation on disease progression can be an important factor for determining the prognosis of disease and for the development of therapeutics, the impact of host genetics should not be ignored due to the complex interaction of host and viral proteins during disease progression.

## Host Genetics Impact on HIV Pathogenesis

### Influence of Human Genetic Diversity on HIV

Previous research has shown that some individuals of European ancestry have homozygous loss-of-function of the C-C motif chemokine receptor 5 (*CCR5)* gene, the only genotype which has consistently associated with protection against acquisition of HIV infection (Samson et al., 1996). Several other genes have been claimed to confer resistance to infection, usually through candidate gene studies, however, these have not been replicated by large GWAS (McLaren et al., 2013). The class 1 human leukocyte antigen (HLA) genes, in particular HLA-B, have been consistently replicated as the major host genetic determinant of HIV viral load and rate of disease progression (McLaren et al., 2015). Similarly, -35 HLA-C variant has been shown to strongly influence spVL and that high HLA-C expression is associated with better control of HIV disease than individuals with lower expression (Thomas et al., 2009). While other genes have been proposed, it is uncertain whether common genetic variants outside of the *HLA* and *CCR5* regions have significant impact on HIV disease progression.

### Effect of Host Genetics on Acquisition

Before the use of GWAS, candidate genes studies were the primary method for identifying genes involved in the acquisition and progression of HIV. These types of studies required understanding of the biological mechanisms of infection, such as gp120 binding to cell surface receptors, for the identification of potential therapeutic or vaccine targets. In a study of HIV-exposed seronegative (HESN) patients in 1996, it was discovered that a 32-bp deletion in the gene CCR5 was able to greatly reduce or prevent infection in HIV-exposed individuals homozygous for the deletion allele (Dean et al., 1996). The CCR5Δ32 variant causes the truncated protein to no longer be expressed on the cell surface (Agrawal et al., 2004) and also associated with reduced disease progression in heterozygous individuals. To date, this deletion variant has not been observed at high frequency in any populations other than Europeans and is the only host genetic variant that has been consistently observed at preventing the acquisition of HIV.

Since the discovery CCR5Δ32, substantial research has been done to identify other genetic variants associated with reduction of HIV susceptibility. Many of these studies have focused on highly HIV exposed seronegative individuals and high-risk populations for identification of susceptibility factors. One large study attempting to determine genetic variants associated with acquisition of HIV examined 848 HIV-negative cases and 531 HIV-positive controls and tested approximately 800,000 SNPs (Petrovski et al., 2011). However, they did not detect any regions which met genome-wide significance following quality control and correction for multiple comparisons.

Recently, a study of the Urban Health Study (UHS) cohort of individuals of African (628 cases and 1376 controls) and European (327 cases and 805 controls) ancestry used a large population size in a high risk population to identify susceptibility variants (Johnson et al., 2015). They reported a region in chromosome 19 which met genome-wide significance (*P* < 5 × 10^-8^) and six other regions which had suggestive significance (*P* < 1 × 10^-6^). However, these results require further investigation as a 2013 study of 6334 patients and 7247 controls of European ancestry was unable to detect any SNPs, outside the major histocompatibility complex (MHC) region of chromosome 6, that met genome-wide significance (McLaren et al., 2013). Thus, the consistent identification of genes that limit HIV susceptibility remains an active area of research.

### Effect of Host Genetics on Viral Load

Upon infection with HIV, the immune system recognizes the presence of the virus through the use of the MHC encoded by HLA genes. These proteins are highly variable and certain *HLA* variants have been implicated in control of HIV infection in diverse populations (Fellay et al., 2009; Pereyra et al., 2010; Leszczyszyn-Pynka et al., 2015). Specifically, *HLA-B* alleles have been identified in several populations as being significantly associated with viral load (Fellay et al., 2009; McLaren et al., 2015). These variants are thought to modify specificity of antigen presentation which can allow differential targeting of HIV-infected cells.

An international consortium study looking at 974 HIV controllers (cases) and 2648 progressors (controls), determined that in European samples (1712 individuals) and African American samples there were no variants that met genome-wide significance outside of the MHC region of chromosome 6 (Pereyra et al., 2010). This study emphasized that the major host genetic determinant of HIV control, in the context of the whole genome, are the *HLA* alleles and *CCR5* genes. When considering the European subset, they identified 313 significant variants all in the MHC region and showed that four of these SNPs explained 19% of the variance in the HIV controller trait.

In a study of African HIV serodiscordant couples from Partners in Prevention HSC/HIV Transmission Study and Couples Observational Study cohorts to determine the effect of host genetics and genital factors (i.e., male circumcision, bacterial vaginosis, or use of acyclovir) of the transmitter on spVL (Mackelprang et al., 2015). *HLA* variants (B^∗^53:01, B^∗^14:01, and B^∗^27:03) and Toll-like receptors (TLR) polymorphisms (TLR2 rs3804100 and TLR7 rs179012) explained 13% and 5% of the variance in viral load, respectively. Other factors, such as plasma HIV levels of the transmitting partner and HLA-concordance between partners were able to explain 10% and 6% of the variance, respectively. Additionally, incorporation of genital factors of the transmitting partner was able to explain 46% of the variation of spVL in this population (Mackelprang et al., 2015).

In the largest spVL genome-wide association study to date including 6315 individuals of European ancestry, it was determined that 24.6% of the observed variability in spVL could be attributed to common human genetic polymorphisms (McLaren et al., 2015). This study again identified *HLA* alleles and *CCR5Δ32* as the only two regions associated with viral load, with no other variants surpassing statistical significance. However, there was a small but measurable contribution (∼5%) from combined common additive effects outside the MHC and CCR5 regions (McLaren et al., 2015).

The MHC region was also observed in a study 538 individuals across three diverse Chinese populations (HAN, YUN, XIN) where it was the only region that met genome-wide significance (Wei et al., 2015). In this study, the authors note that, although the same region has been implicated in other populations (European and African American), the identification and significance of variants varied greatly. The authors proposed that this is because linkage patterns between tagged SNPs and causal variants may differ per population, and that there may be different causal variants in these Chinese populations compared to Europeans and African populations, and/or minor allele frequency in the populations may result in different associated SNPs (Wei et al., 2015).

Taken together, these studies show that host genetics can explain roughly 30% of variance in viral load, however, a majority of the remaining variance is still unknown but is thought to be environmental factors and/or unidentified host factors. Therefore, to better understand variation in HIV disease progression related to spVL, more research is needed in larger samples using novel analytical methods that provide more power for detecting smaller genetic effects and additional ancestry groups for identifying non-European variants.

## Characterizing the Host–Pathogen Interaction Through HIV Host Dependency Factors

In order to understand the pathogenesis of HIV infection, it is important to explore how viral and host proteins interact. HIV only encodes nine genes and requires the use of host proteins to establish and maintain infection, termed HIV host dependency factors (HDFs) (Brass et al., 2008). Generally, there have been two different genome-wide methods employed to identify these interactions including: genome-wide siRNA knockout screens and more recently genome-wide CRISPR knockouts. These methods have identified multiple HDFs, however, the precise pathways involved often differ, possibly dependent on the specific methods used.

RNA-interference (RNAi) based studies are popular for identifying large numbers of HDFs required for establishing and maintaining infectious diseases and three initial studies proposed over 800 HDFs required during HIV infection (Brass et al., 2008; König et al., 2008; Zhou et al., 2008). The first RNAi-based HIV HDF study aimed to characterize HDFs involved in pathogenesis through two different screens (Brass et al., 2008). The first screen used HIV-IIIB to identify host proteins involved with viral entry and Gag translation but was unable to identify proteins involved in viral assembly and budding. This was subsequently addressed by a second screen performed in HeLa-derived TZM-bl cells, expressing transgenic CD4 and CCR5, to identify factors involved in viral assembly and budding (Brass et al., 2008). While HeLa cells are not physiologically relevant to HIV pathogenesis, they provide insight into some potential cellular functions that can be replicated or confirmed using primary CD4+ T cells.

Shortly after, König et al. (2008) used a similar RNAi method to determine host factors associated with early infection (König et al., 2008). This study identified 295 genes and, when compared to the 283 genes determined by Brass et al. (2008), discovered 13 genes that were statistically significant in both screens (Brass et al., 2008; König et al., 2008). The difference may be due to the different cell lines used between the studies or variation of the lentiviral vector used for transfection of the study (Table 1).

**Table 1:** Summary of RNAi and CRISPR-Cas9 genome-wide knockout experiment characteristics for the study of HIV disease.

Study (Year)	Type of screen	Cell types^1^	HIV-1 strains^2^	Reference
Brass et al., 2008	RNAi	HeLa-derived TZM-bl cells	HIV-1 IIIB	Brass et al., 2008
König et al., 2008	RNAi	HEK-293 T cells	HIV-1 VSV-G	König et al., 2008
Zhou et al., 2008	RNAi	HeLa P4-R5	HIV-1 HXB2	Zhou et al., 2008
Zhu et al., 2014	RNAi	HeLa MAGI	HIV-1 IIIB	Zhu et al., 2014
Park et al., 2017	CRISPR	GXRCas9	HIV-1 JR-CSF	Park et al., 2017

*^1^Cell types used for RNAi or CRISPR screening. ^2^HIV-1 strains used for RNAi screens.*

Zhou et al. (2008) used β-gal activity after 48 h, to identify host factors associated with viral entry, or at 96 h to identify factors responsible at all stages of infection (Zhou et al., 2008). Of the 232 HDFs identified in this screen, 15 overlapped with Brass et al. (2008) (Zhou et al., 2008). Although, limited, it was determined that this overlap was higher than what would be expected by chance alone. The authors acknowledged that the differences between genes identified in their study and the previous Brass et al. (2008) study may be due to transfection time, type of reporter (Tat-driven or p24), and/or the nature of the algorithm-generated siRNA libraries (Brass et al., 2008; Zhou et al., 2008). While these screens have little consistency of identified genes, they do detect genes within the same biological processes. For example the SP1/mediator complex and the NF-κB signaling pathways (Zhou et al., 2008). Subsequently, a meta-analysis of these three screens determined that there was significant functional overlap of the implicated genes at the pathway level, implicating Nuclear Pore/Transport, GTP Binding, Protein Complex Assembly, and DNA repair (Bushman et al., 2009). These studies propose that ∼9.5% of human protein coding genes are now implicated in HIV replication (Brass et al., 2008; König et al., 2008; Zhou et al., 2008; Bushman et al., 2009).

In a more recent study by Zhu et al. (2014), they used Multiple Orthologous RNAi Reagent (MORR) screens as well as an RNAi Gene Enrichment Ranking (RIGER) method in order to minimize false positives and negatives (Zhu et al., 2014). This study identified c3orf58 (renamed GOLGI49^∗^), SEC13, COG, and THOC2 as key HDFs and characterized the roles of these genes *in vitro.* Notably, GOLGI49 was identified as a Golgi protein and during knock-down shown to decrease replication of both HIV IIIB (X4-tropic) and BaL (R5-tropic) viruses (Zhu et al., 2014). THOC2, and by association the THO/TREX complex, was identified as a potential key complex involved in regulation of HIV replication, however, more studies are needed to determine the mechanism of action (Zhu et al., 2014). SUPT16H was identified by the RNAi screen and later confirmed to play a key role in HIV transcription (Zhu et al., 2014; Huang et al., 2015). SEC13 was determined to play an essential role in viral replication prior to viral integration but after nuclear import in both Jurkat and primary CD4+ T cells.

Although strategies like MORR-RIGER and Genome-wide Enrichment of Seed Sequence (GESS) analysis can reduce false positives and off target effects, further techniques and standardizations are required. There is currently no standard cell line for HDF work with groups which may explain the variable results observed in these studies. HeLa-derived cell lines and MAGI cells require the addition of CCR5 to become susceptible to R5-tropic HIV infection which strains their physiological relevance. Therefore, without consistent use of cell-types, the physiological landscape of protein expression may cause increased variation and further deviation from the expected in primary cell lines.

Recently, genome-wide CRISPR knockouts screens have become more popular for generation of loss-of-function variants as they have increased knock-out reliability and decreased off target effects compared to siRNA methods (Shalem et al., 2014; Wang et al., 2014). This technology allows for targeting of various host cells with increased efficacy to address concerns that RNAi based screening can allow for low-level protein expression. Indeed, it has been shown that the use of CRISPR-Cas9 lentiviral single-guide RNA constructs can achieve greater specificity and sensitivity than the use of RNAi-based screens previously allowed (Park et al., 2017).

In a genome-wide CRISPR knockout study, novel host–pathogen interactions involving *TPST2*, *SLC35B2*, and *ALCAM* during HIV pathogenesis were identified, not seen by the previous RNAi-based screens (Park et al., 2017). They showed infection inhibition in primary human CD4+ T cells supporting these factors as key genes in HIV infection with loss-of-function variants without impairing cell viability. *TPST2* and *SLC35B2* were shown to be involved in sulfation of CCR5 on extracellular tyrosine residues (Park et al., 2017). Knockdown of TPST2 and SLC35B2 prevented proper extracellular folding of CCR5, thereby inhibiting interaction with viral gp120. While the role of *ALCAM* is not fully understood, Park et al. showed it was required for effective cell-to-cell transmission of HIV. Importantly, the genome-wide CRISPR knockout approach of Park et al. can be modified for use of studying entry of other viruses (Schott and König, 2017).

While CRISPR and RNAi technology has identified large numbers of potential HDFs in model systems, the relevance in humans is still unclear. While cell lines may be able to survive with a particular gene knockdown, whether the gene is essential for human life cannot be determined from the present screening data. However, the Genome Aggregation Database (gnomAD)^a^, has over 15,000 whole-genome and over 120,000 exome sequences which can be used to identify individuals with predicted homozygous loss-of-function alleles. If an individual is healthy with a homozygous loss-of-function allele, the gene is likely not essential for human life and may make a valuable drug therapy target.

## Conclusion

It has been clearly demonstrated that both host and viral genetics play a vital role in determining HIV acquisition and disease progression. Current evidence supports the role that viral subtype has an effect on disease progression, however, there is also evidence against this claim. Overall, viral diversity has been demonstrated to have an impact of disease progression and therefore, it is important to study disease progression in the context of both host and viral genetics.

As GWAS have become a useful tool for discovering novel variants associated with a particular trait, it is important to recognize that their use is limited by the diversity of the target population. In 2016, it was reported that “genomics is failing on diversity” with the vast majority (81% over 2511 studies) of GWAS, in all disciplines, being performed in individuals of European ancestry in 2016 (Popejoy and Fullerton, 2016). These tools have not been used to their full potential and replication of studies within large, diverse populations may yield novel associations. While GWAS of other phenotypes have benefitted from cohorts of over 100,000, the largest cohort of HIV was only 6315 individuals. Larger sample sizes are required for the detection of genetic variants outside of the MHC and CCR5 regions, and this will require additional investment in developing new and diverse cohorts and acquiring the required clinical and genetic data.

Genome-wide knockout studies have great potential for studying the effects of HDFs for a variety of infectious diseases; however, translation of their physiological relevance in primary cells or *in vivo* remains an area of active research. Cross-referencing the genes from RNAi and CRISPR genome-wide studies with large databases such as gnomAD for identification of genes which have homozygous loss-of-function in individuals of various populations could provide interesting avenues for new therapeutics. This is akin to reading the *natural experiment* where healthy individuals with a homozygous loss-of-function gene purported to be necessary for HIV replication could act as valuable resources to determine the gene’s action *in vivo*.

As the field of genomics continues to evolve, there is opportunity to leverage the growing wealth of information available to better understand disease acquisition and progression.

## Author Contributions

RT and PM conceived and wrote the manuscript.

## Conflict of Interest Statement

The authors declare that the research was conducted in the absence of any commercial or financial relationships that could be construed as a potential conflict of interest.
